# From Contact to Conversation: Protactile Language, Modality, and Community

**DOI:** 10.1146/annurev-linguistics-011724-121536

**Published:** 2025-10-13

**Authors:** Deanna L. Gagne, Hayley Broadway

**Affiliations:** 1 Department of Linguistics, Gallaudet University, Washington, DC, USA; 2 Protactile Kids (PT Kids) Lab, Gallaudet University, Washington, DC, USA

**Keywords:** protactile, DeafBlind, tactile modality, language emergence, typology, positionality

## Abstract

Protactile language, a tactile language that has emerged within the DeafBlind community in the United States over the past two decades, challenges conventional assumptions about language, modality, and communication. Originating from a grassroots movement to center touch as a valid epistemology, protactile has developed distinct linguistic structures grounded in contact space, reciprocity, and embodied intersubjectivity. This article reviews the emergence and linguistic development of protactile, highlighting key structural innovations and areas of ongoing research. We discuss how protactile reconfigures foundational concepts such as phonology and interactional structure. We also present ethical considerations involved in studying a community-based language—emerging or otherwise—and emphasize the need for research practices grounded in collaboration and accountability. By centering the tactile experience and DeafBlind lived experiences, protactile contributes to a broader understanding of human language, how it functions, and how it emerges within diverse sensory and cultural ecologies.

## 1. INTRODUCTION

In 1960, with his seminal piece “Sign Language Structure: An Outline of the Visual Communication Systems of the American Deaf,” William Stokoe [2005 (1960)] proposed a revolutionary idea: Linguistic structures can exist in modalities other than speech. Before this, the manual productions of deaf people were thought to be either, at best, visual analogs of spoken language or simply gesturally iconic productions without the usual hallmarks of true “language.” Indeed, the proposal was met with mixed reviews, both within the deaf community and in the field of linguistics (Hochgesang & Miller 2016). Regardless of its initial reception, the notion took hold that American Sign Language (ASL) and other sign languages worldwide were indeed languages in their own right and did not take their value, or linguistic elements, from spoken languages. Since then, research on sign languages has introduced numerous new possibilities to the field of linguistics and related fields, such as psycholinguistics and the cognitive sciences.

Here we review a brief history of a new language in a new modality (touch), aptly named protactile^1^ by its community of users.^2^ We review recent linguistic findings regarding protactile and follow with a discussion of possible questions to be asked about protactile, given the introduction of a new modality of human language. We end with a discussion of ethical considerations, as the study of protactile has spurred interest within the linguistic community.

## 2. HISTORY AND EMERGENCE OF PROTACTILE

Protactile is a new language that got its start in the DeafBlind^3^ community in the United States in 2007 (granda & Nuccio 2018). Several works have since detailed the rise of protactile (e.g., Clark & Nuccio 2020; Edwards 2014, 2018); readers are encouraged to seek out these publications for a more detailed understanding of the conditions under which protactile emerged. Here we offer a snapshot of the history of protactile and some of the recent linguistic findings regarding its linguistic structures and use.

Protactile emerged naturally among DeafBlind individuals concentrated in Seattle, Washington, in 2007. Up to that time, when participating in meetings, the communication of DeafBlind individuals was almost always mediated by interpreters regardless of whether the conversation was with sighted individuals (hearing or deaf) or even other DeafBlind individuals. Typically, two interpreters would be assigned per DeafBlind individual for their communication needs (e.g., Clark 2023). However, per ASL interpreter standards, they were not expected also to accompany or guide the DeafBlind individual to, say, the bathroom, or to get food when there was a break in a long meeting; that would be done by yet a third person called a Support Service Provider (SSP). Therefore, for each DeafBlind individual, there would be, on average, two to three people providing “access” to the meeting and the physical environment. However, in 2007, something changed: One organization serving DeafBlind individuals, the DeafBlind Service Center (DBSC), underwent a change in leadership, hiring several DeafBlind individuals to manage operations (granda & Nuccio 2018). This hire marked the first time that DBSC had a DeafBlind director. This fact attracted more DeafBlind individuals to work there. Eventually, there were so many DeafBlind individuals participating in meetings and doing their work that the organization simply could not find enough interpreters or SSPs to meet the needs of its employee base. They decided to do something radical: go without interpreters and directly communicate with each other.

This simple decision was revolutionary. Indeed, the protactile movement constituted more than just a shift in language; it originated as a shift in thinking—a shift in interacting and in being in the world that manifested in linguistic changes. The belief was, and still is, that DeafBlind individuals should not need to excuse themselves for using touch to explore and engage with the world. Rather than expecting them to conform to norms shaped by sighted and hearing experiences, there was a growing recognition of the value in creating environments that honor and support tactile ways of being (Clark 2023, Edwards 2024, granda & Nuccio 2018).

An important thing to note is that the DeafBlind individuals participating in the protactile movement were often already bilingual in ASL (a visual–manual language that originated in the sighted–deaf American community) and, to varying degrees, English. However, neither ASL nor English (written or spoken) was readily understood by DeafBlind individuals, which makes sense—DeafBlind individuals created neither of these languages. Up to this point, they had been adapting ASL in various ways to shoehorn the visual language into a tactile modality, but with variable success (see granda & Nuccio 2018). This was partly because of the interpreters; interpreters were trained primarily in ASL and were providing Tactile ASL access, which was not always accessible. For example, the signs for MOTHER^4^ and FATHER are distinguishable in ASL due to their placement on the signer’s body—either at the chin or at the forehead. These are different signs in visual ASL and are readily perceptible to sighted people, who can see the relative location of the hands to the head. However, if a DeafBlind person were tracking the production of these signs in isolation (e.g., in the sentence “My father likes bears”), it would be difficult to perceive whether the hand had been placed at the chin or the forehead, especially without touching the signer’s head with the nontracking hand simultaneously and noting the relative relationship between the hand and the head (Gagne et al. 2023). Note that touching another person’s face without explicit permission is unacceptable and rare, even with permission from the DeafBlind person. Other complex handshapes in ASL are also difficult to perceive using touch; for example, the hand configurations (handshapes) for the numbers seven (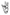) and eight (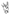) are practically identical, even visually (e.g., Berteletti et al. 2021), and are even harder to distinguish when trying to feel these productions. Finally, Tactile ASL was not working because so much of what is conveyed in ASL (or other sign languages) happens on the face. When DeafBlind individuals are interacting with someone using Tactile ASL, the message is rendered “flat”; they miss critical grammatical and affective information that is expressed through facial expressions, such as tone, emotion, and question marking (for a review of the role of nonmanual expressions in ASL, see Wilbur 2021).

### 2.1 Protactile Principles

Given the issues with Tactile ASL, and then excusing interpreters from their daily communicative interactions, the DeafBlind individuals in Seattle began to reflect on the things that they cared about, what worked, and what did not. This led to the development of the protactile principles. Today, the principles continue to be reevaluated, and they too evolve as the language and its community of users grow. These principles uplift the DeafBlind experience by centering the tactile approach, acknowledging what is and is not informative to a DeafBlind person and bringing to the forefront a shared, embodied way of knowing that prioritizes touch over sight.

Readers are encouraged to review the full list of protactile principles and their history (granda & Nuccio 2018), but a few are highlighted here.

#### 2.1.1. Contact space.

**Figure 1:**
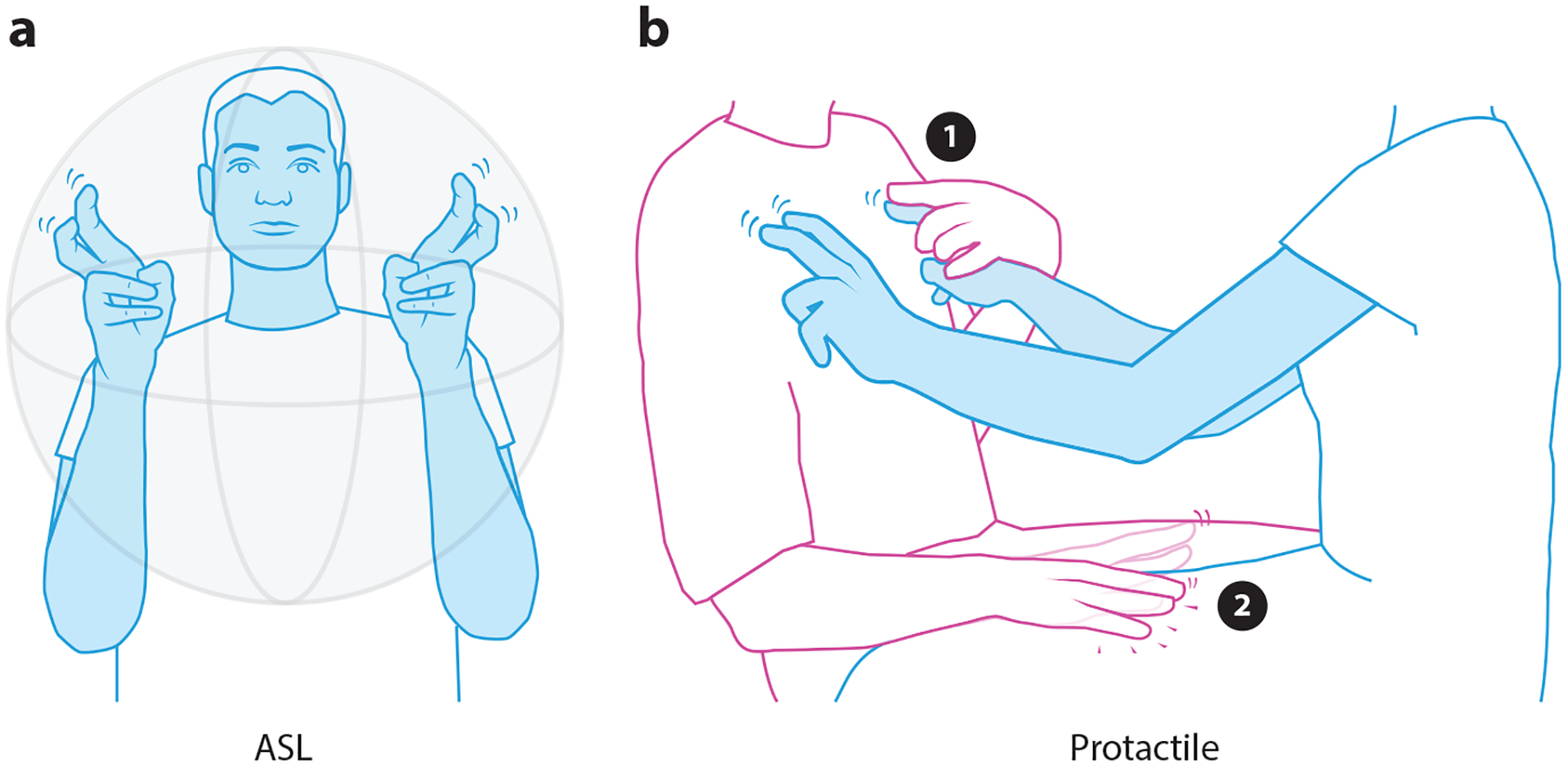
Side-by-side comparison of the production of the concept of a “title” in (*a*) American Sign Language (ASL) and (*b*) protactile. (*a*) In ASL, this sign is produced in air space, demonstrated by the invisible sphere. (*b*) In the protactile example, the speaker has borrowed this sign (see Section 2.2 for a discussion of borrowing) and adapted it to follow the principle of contact space by producing it on the listener’s upper chest (①) (see Section 2.1.1). The listener lets the speaker know they are following along by tapping their hand on the speaker’s leg at regular intervals (②). This backchanneling is discussed in Sections 2.1.2 and 3.2. Graphic design by Silvia Palmieri.

Contact space is central to the protactile principles. It refers to the use of the listener’s body as the primary site for communication, rather than relying on air space as visual signed languages do (Figure 1). All spatial information, such as reference points, role shifts, and emphasis, is expressed through direct, tactile contact with the listener’s body. Air space is the noncontact space that is generally in front of the body. Using contact space ensures that communication is accessible, intuitive, and grounded in the tactile experience. In (visual) ASL, the speaker moves their hands through air space. The speaker can point to a particular area in front of their body, for example, as a referential point. For example, someone discussing their friend Juan may point to a place, and sign Juan’s name, associating Juan with that location. Then, the speaker can later point to that place, using it as a pronoun, meaning “he/Juan” (e.g., Cormier et al. 2013, Meier & Lillo-Martin 2013), or produce signs to and from that location in air space, indicating grammatical relationships between referents, such as subject and object (e.g., Lillo-Martin & Meier 2011, Wilbur 2013). However, air space is very difficult to track for someone who is DeafBlind. Instead, they will use contact space, establishing referents by touching the listener’s body, such as tapping a shoulder and establishing that location as “Juan.” Then they can direct movement to and from that contact point to indicate grammatical relationships (see descriptions in granda & Nuccio 2018). These referential contact points can also be produced along the listener’s thigh, arm, or upper chest. Comfort with touch varies by person and context. Protactile norms guide respectful negotiation of contact space and articulator placement—not to prescribe rigid rules but to support the co-creation of dialogue and to resist hearing, vision-centric distancing.

Another example of contact space is in the use of classifiers in ASL. In ASL, specific handshapes such as 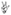 and 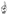 (for vehicles and people, respectively) serve as classifiers, defined generally across languages as morphological elements, either words or affixes, that typically co-occur with nouns serving to categorize referents based on features such as animacy, shape, sex, and so on (see, e.g., Aikhenvald & Mihas 2019, Allan 1977). In ASL, these handshapes are moved through air space to depict motion or spatial relationships—for example, a person walking erratically or a car driving up a hill. In protactile, rather than using air space, the speaker enlists the listener’s nondominant hand to represent these entities, then acts upon the hand to produce concepts of size, shape, action, and experience (Edwards & Brentari 2020, van der Mark 2023). For example, the ASL classifier for a person, 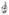, is replaced in protactile with an expression denoting the legs (a downward facing 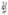). A person walking from place to place in ASL would move through air space from point A to point B. In protactile, the legs walk across the listener’s arm or leg to express the same concept. This change allows for better contact between the most physically perceptible part of the hand—the fingers as legs—while specifying the referent as a person. In this way, ASL signs that were difficult to perceive were replaced with new productions that were highly perceivable and distinguishable in the tactile modality.

#### 2.1.2. Reciprocity.

Protactile is fundamentally reciprocal. All participants, regardless of their vision status, are expected to engage through touch. This principle ensures that DeafBlind individuals are not passive recipients of visually dominated communication but rather full participants in a shared tactile discourse. Reciprocity disrupts asymmetrical access and reorients communication around the tactile norm (granda & Nuccio 2018). An example of reciprocity in action is when a sighted individual and a DeafBlind individual are in conversation. Sometimes, the sighted person may use protactile or Tactile ASL with the DeafBlind person, but the DeafBlind person, knowing they are signing with a sighted person, may still sign ASL in air space. Moreover, the sighted person may not think to put their hands on the DeafBlind person’s hands to show they are listening/attending to the message. In this situation, without touching the DeafBlind person in the ways offered by the protactile principles, the sighted person does not have a good way of backchanneling to show agreement, confusion, or even just that they are following along. The DeafBlind person may ramble on in what is essentially a monologue, out of alignment with the addressee. This highlights how protactile is reciprocal in ways that other tactile communication methods are not. Most of these other communication methods, such as Tactile ASL and Haptics (e.g., Raanes & Berge 2017), are intended for the DeafBlind person to primarily be a passive recipient of the information and not necessarily an equal contributor to a conversation. Taking the same situation, if both interlocutors were following the protactile principles, whether they were sighted or not, they would have one hand “listening”to the other’s message on the speaker’s dominant hand, and their other hand would provide reciprocal feedback on the speaker’s nondominant arm or leg (see Figure 1). Some specifics of backchanneling are individual to each person and are somewhat intuitive as the listener expresses their responses such as surprise (a squeeze or a hard tap), laughter (quick light scratches), questioning (tracing a question mark), general nodding (paced tapping), and so on.

#### 2.1.3. Information source.

The information-source principle emphasizes the need to make the origin of information clear. In visual contexts, people often rely on visually accessible environmental cues or background knowledge to infer where information comes from. In protactile environments, these cues must be made explicit through touch. This includes physically incorporating the source into the conversation, such as guiding the listener’s hand to the device, person, or object where the source was obtained, or explicitly naming it (granda & Nuccio 2018).

For example, imagine two people on a bench, both DeafBlind or one sighted and one DeafBlind. One receives a text saying that a third friend, Isabella, is on her way. Rather than saying, “Isabella is on her way” without any additional information, the person who received the text could guide their interlocutor’s hand to the phone (or device) and say, “I just got a text from Isabella. She says she is on her way.” Without the information about the source of the knowledge, the other friend may wonder: Did they see Isabella? Did they previously agree on a time? Is it just a guess? Explicitly identifying the source ensures a shared understanding and supports trust between participants.

In sum, the protactile principles created an equitable, immersive, and embodied framework for communication that centers the DeafBlind experience and fosters full access through touch. It was this framework that led to the further emergence of protactile, the language.

### 2.2 Emergence of Protactile Vocabulary

Protactile’s core vocabulary is still growing (Clark & Nuccio 2020, Edwards & Brentari 2020). Communication between DeafBlind adults includes a combination of core protactile vocabulary, foreign (borrowed) vocabulary, and spatial language. Core words are those that are particular to protactile and resemble neither of the other languages known to DeafBlind adults in the United States: English or (tactile or visual) ASL. An example of newly developed core words in protactile would be in reference to “mother” and “father.” As mentioned earlier, the ASL signs for MOTHER and FATHER are distinguishable only by the placement relative to the head, with MOTHER placed at the chin and FATHER placed at the forehead. The distinction between the two is very difficult for a DeafBlind person, who cannot simultaneously perceive the head to understand where the hands are being placed (see, e.g., Clark 2023). Over a short period of time, protactile users came up with a new way to express “mother” and “father”: “Mother” is produced by pulling the fingers together from a 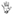 to an 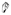 on the listener’s body (on the arm, leg, upper chest—the location does not contribute to the meaning^5^), while “father” is produced by starting with the fingers closed, going from 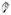 to 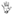 This way, the concept of “parents” can be produced by putting the two in sequence: [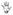 - 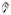 - 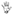]. Borrowed vocabulary is just that—vocabulary borrowed from either ASL or English print. These words serve the purpose of filling the temporary lexical gaps in protactile to keep the conversation going even though they may not be as readily perceptible as the core protactile vocabulary would be. An example of borrowed vocabulary from ASL is displayed in Figure 1, where the ASL sign for “title” is adapted from air space to contact space. Over time, it is possible that protactile users needing to refer to this concept frequently may come up with a new, core production and do away with this borrowing altogether. In fact, we have observed a different way to refer to the concept of titles already, but we are not sure if this new production will become commonplace across protactile users. Spatial language involves those productions that are part of the core productions in protactile but are not fixed vocabulary items. They may include some iconic descriptions—for example, of the girth and texture of a tree or of the function of a tool.

ASL and other languages work similarly by having a combination of core and borrowed vocabulary; signers or speakers may borrow from other languages that they know to fill gaps in their vocabulary (see, e.g., Gawlitzek-Maiwald & Tracy 1996,Tulloch & Hoff 2023). The main difference is that protactile, as an emergent language, has many current gaps, but these gaps are being filled rapidly through the generation of new core lexical items. In the next section, we review some of the research regarding protactile and some new innovations that have emerged in the last few years.

## 3. PROTACTILE RESEARCH

We note that, given its recent emergence, there are few published research studies of protactile. Here, we summarize the existing research and discuss possible future directions for protactile research.

### 3.1 Phonology of Protactile

In their article “Feeling Phonology: The Conventionalization of Phonology in Protactile Communities in the United States,” Edwards & Brentari (2020) investigate the emergence of protactile’s novel phonological system. As touched upon earlier, protactile is produced in contact space, which requires two people, or essentially, four hands (two hands/articulators per person). Edwards and Brentari focus specifically on “proprioceptive constructions,” which are the tactile counterparts of classifier constructions in visual sign languages. They show how specific linguistic functions (e.g., initiation, object representation, movement, continuity prompts) are systematically distributed across the four articulators. They argue that this distribution signals the emergence of phonological organization within the tactile modality.

They further argue that the shift from air space to contact space is not merely an adaptation of ASL but is a foundational reorientation toward a new linguistic system. This evidence also supports a broader definition of phonology independent of sensory modality and demonstrates how linguistic structures can emerge under a novel sensory and communicative approach. It is commonly asked whether protactile is simply another “signed” system. While it is true that there is an overlap in articulators for most visual–signed systems and protactile (i.e., the arms/hands), the perception of protactile is completely different. Protactile is a language that is produced with the arms and hands but is perceived using the tactile channel—in other words, the body (Edwards 2014, 2024; Edwards & Brentari 2020, 2021; Lu et al. 2024). Signed languages are also produced with arms and hands but are organized around visual perception. The phonological reorganization in protactile lays the groundwork for broader grammatical development, such as the emergence of new deictic and interactional systems, as discussed below.

### 3.2 Emergence of Demonstratives in Protactile

Building on their analysis of phonological organization, Edwards & Brentari (2021) follow up with a study on how demonstratives have become grammatically integrated into protactile language. They identify four distinct types of “taps”that emerged in the language: backchanneling taps; two types of demonstrative taps (exophoric and endophoric); and “propriotactic taps,” a novel form that coordinates the four hands involved in a protactile interaction.

The four types of taps identified by Edwards & Brentari (2021) serve distinct yet interrelated functions. Here we give examples all within a single interactional context: giving cooking instructions. Backchanneling taps are produced by the listener to indicate continued attention or agreement, helping sustain mutual engagement. For example, as the speaker provides step-by-step instructions for preparing a dish, the listener may lightly tap the speaker’s lap to show they are following and ready for the next step (see Figure 1). Exophoric demonstrative taps are used by the signer to direct attention to an object in the shared physical environment, such as tapping a bowl to indicate where to put the next ingredient. Endophoric demonstrative taps refer back to elements already established in the discourse. For example, after establishing three tactile representations of cooking steps along the listener’s arm, the speaker may tap the second one to focus attention on that part of the process. Finally, propriotactic taps initiate coordinated articulation by signaling that the listener’s hand should take up a particular shape, perhaps an upward facing 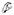 so they may coordinate the production of the concept of stirring ingredients in a bowl. Each type of tap serves different communicative and grammatical functions, but they share an underlying role in modulating intersubjective attention, which Edwards & Brentari (2021) argue is central to the emergence of a tactile deictic system.

Edwards & Brentari (2021) further argue that the development of demonstratives in protactile reflects a broader grammaticalization of intersubjectivity rather than spatial deixis per se. As mentioned above, since protactile language is rooted in contact space, as opposed to the air space used by visual sign languages such as ASL, it challenges and reconfigures traditional assumptions about articulation, perception, and linguistic grounding. It also contributes to our understanding of language emergence by highlighting how attention-directing and modulating interactional cues (like backchanneling taps) can evolve into grammatical markers in a tactile modality. These findings not only expand on our understanding of deixis in a tactile language but also underscore the centrality of intersubjectivity in shaping grammatical systems. Given such structural and functional innovation, it is natural to ask how protactile is processed in the brain, recruiting language-related areas or otherwise.

### 3.3 Protactile Recruits Typical Language-Related Neural Networks

One major question regarding protactile is whether its speakers process it neurologically as a language. In her dissertation, Lauren Berger (2021) used functional near-infrared spectroscopy to investigate whether the brain’s sociocommunicative and language networks function independently of sensory modality. Her study compared DeafBlind protactile users with deaf–sighted ASL users in interactive communication contexts. Berger found that both groups recruited canonical left-hemisphere language regions during communication regardless of whether the language was conveyed through touch or vision. Further, both groups showed evidence of embodied cognition during language reception, and DeafBlind participants’ mutual touch activated similar brain regions as mutual gaze did in sighted individuals. Berger argues that these findings suggest that the brain’s language and social engagement systems are modality-independent and that protactile could function as an early first language for DeafBlind children, supporting early linguistic development regardless of the language modality from the outset. Interestingly, as noted above, all adult protactile users are multilingual and multimodal to varying degrees, with language repertoires that typically include protactile, ASL, and English—protactile being the most recent addition. As such, it is possible that the neural activation observed in Berger’s study reflects the interaction of these languages rather than the isolated effects of protactile alone. Nevertheless, the neurobiological processing of a language grounded in touch remains an open and interesting question for future research.

### 3.4 Protactile Acquisition in DeafBlind Children

One unique feature of protactile is that it is a language that has emerged among adults, without interaction with or involvement of children. This is very different from what has been documented across most emerging sign languages, which have children as the primary contributors to a given language’s emergence (for a review of emerging languages, see Meir et al. 2010; for a closer look at the emergence of Nicaraguan Sign Language, see Senghas et al. 2014). Can DeafBlind children acquire protactile in the same way that children acquire other natural languages? And how might their participation further shape the language as it continues to emerge? We and our team of collaborators have begun to investigate what happens when DeafBlind children are exposed to protactile from an early age. Theoretically, as a language, protactile should be acquirable by children, and in fact, children may contribute to further change in the language through at least two processes: by introducing new structures themselves or by provoking change in others (e.g., adults) who are interacting with them. First, there is strong evidence to suggest that children themselves contribute to language change over time. Across every generation, children introduce new structures and ways of using existing structures inherited from previous generations (e.g., Labov 2011, McWhorter 2001, Newport 1999). This change can happen quite rapidly in some cases, such as in Nicaragua, where in the 1980s and 1990s, younger children introduced new grammatical structures to the language they learned from older peers (e.g., Polich 2005, Senghas 2003, Senghas & Coppola 2001).

In addition to innovating themselves, children can provoke linguistic change in adults by requiring adaptations to meet their developmental and communicative needs. Adults frequently simplify, restructure, or reframe their language in response to children’s attention patterns, comprehension strategies, and interactional behaviors (see, e.g., Cameron-Faulkner et al. 2003, Rowe 2012 for non-Western contexts, see, e.g.,Casillas et al.2021). For instance, English-speaking caregivers often rely on familiar, partially schematic constructions when speaking to young children, reflecting adaptations to children’s limited processing and early production (Cameron-Faulkner et al. 2003). Similarly, in a small-scale community in Papua New Guinea, caregivers adjust their speech based on children’s engagement, responding more when children vocalize or seek interaction (Casillas et al. 2021). These kinds of adjustments reflect a broader, cross-cultural pattern in which adults modify their language to scaffold children’s participation in interaction, potentially shaping the structure and use of emerging languages over time.

Indeed, Lu et al. (2024) further illustrate the role of children in shaping adult language and communicative behavior in DeafBlind interactions. In their study, a DeafBlind educator engaged in extended home-based sessions with two DeafBlind toddlers over several months. The DeafBlind educator adapted her use of protactile taps, touch-based strategies, and environmental structuring in response to the children’s sensory access, attention patterns, and modes of exploration. Instead of introducing the children to a set of established vocabulary, she prioritized reciprocal tactile engagement, modifying her use of communicative tools, scaffolding the children’s understanding and participation. These adaptations evolved as the children responded and evolved themselves, revealing emerging competencies or changing attention. The DeafBlind educator did not simply model protactile but coconstructed it with the children, reinforcing the idea that young learners can actively shape both the form and delivery of linguistic input through their responses and needs.

To explore these dynamics, we (Gagne et al. 2023) conducted a parallel case study of a DeafBlind toddler also interacting regularly with a DeafBlind protactile educator. Over a 4-month period, the adult visited the child’s home twice a week for play-based sessions, during which their interactions were recorded. We then analyzed the recordings to determine whether the child would begin to acquire protactile productions and the ways in which the adult structured their tactile input. In 13 minutes of video sampled from the final month of data, we documented over 300 distinct touch events, averaging more than 24 distinct tactile events per minute. We further showed that the child increasingly engaged in meaningful contact, including the production of an attention-modulating tap (propriotactic taps; cf. Edwards & Brentari 2021). These findings suggest that even with limited exposure, a DeafBlind child can begin to acquire and use protactile signals in developmentally appropriate ways.

We also explored the nature of the tactile interactions between the DeafBlind adult and child. As reported in our study (Gagne et al. 2023), the child showed increasing willingness to engage in prolonged contact using nonactive body parts. For example, he rested the sole of his foot against the adult’s thigh as they sat on the floor and engaged with toys using their hands. We interpret this to mean that the DeafBlind adult’s interactional style, which is grounded in the tactile orientation and uses appropriate protactile practices, may have differed significantly from the ways in which sighted individuals typically interact with DeafBlind children. Sighted people may inadvertently initiate touch in ways that are abruptive and disorienting with a tactile frame of reference. Sighted people have implicit, subconscious ways of maneuvering through the world that also shape how they interact with others. When approaching someone, they often assume—without realizing it—that the other person can see them coming and is prepared for the interaction. This assumption breaks down when approaching a DeafBlind person. For example, a sighted person may grab a DeafBlind person’s hand or arm without warning, which, to the DeafBlind person, is abrupt and disorienting. In protactile, the approach is different. The person initiating the interaction places a hand on the DeafBlind person’s upper arm or shoulder—a more neutral body part—and then traces down the back of the arm to the hand to begin the exchange. This method signals presence and intent, giving the DeafBlind person time to orient and the autonomy to choose whether to engage. DeafBlind people, whose experiences are grounded in touch, often engage in tactile interactions with greater ease and mutual understanding, approaching one another with shared assumptions.

We suggest that over time, DeafBlind children’s negative experiences with the way that sighted people approach them may contribute to what has been documented as touch aversion (Costain 2020). Note that the touch aversion we have observed in DeafBlind children seems to be directly related to human touch, and not to touching objects. In contrast, the DeafBlind adult in our study often approached the child with passive, coregulated touch (e.g., shoulder-to-shoulder, leg-to-leg, back of her hand to their arm or leg), which may have supported the child’s comfort and participation in subsequent active tactile interactions. This behavior suggests the early emergence of tactile copresence, not only through directive, communicative touch but also through passive, grounding forms of physical contact. These observations align with Vatanen’s (2021) concept of comfortable silences in spoken discourse, suggesting that passive tactile contact can play an analogous role in maintaining connection and mutual orientation in protactile contexts.

These findings align with theoretical work by Edwards (2022), who shows how the failure to adapt communicative strategies to the tactile orientation of DeafBlind individuals can result in misalignment, inaccessibility, and disempowerment. When sighted norms—such as pointing to unstructured air space or relying on ASL fingerspelling—are imposed on DeafBlind interlocutors, the resulting interactions can create confusion and dependence on sighted intermediaries, such as the interpreters and SSPs mentioned above. In contrast, protactile engagement, grounded in shared experiences and understanding of tactile space, allows DeafBlind individuals to experience and act on information autonomously. This supports our observation that DeafBlind children are more likely to remain engaged when approached using tactile-oriented, passive forms of touch, thereby laying the foundation for copresence and language development. This also underscores the value of involving DeafBlind collaborators in studies with the DeafBlind population, a topic we discuss below.

## 4. FUTURE DIRECTIONS

As protactile language continues to grow, it offers a rare and invaluable opportunity to examine how language emerges, evolves, and is shaped by the tactile modality. Drawing on what we have learned from the growing body of literature on visual languages such as ASL (e.g., Emmorey 2023, Sandler & Lillo-Martin 2006), which also involves a shift in thinking about the interaction between language and modality, we can begin to outline possible future directions in the study of protactile.

One avenue is the investigation of how the tactile modality interacts with perception and cognition. Just as deaf signers show enhanced visual–spatial attention (e.g., the inferior visual field) due to early and sustained exposure to a visual language (Stoll & Dye 2019), DeafBlind individuals using protactile may develop heightened tactile–perceptual abilities in specific areas of the body (for a parallel example of visual processing from ASL, see Caselli et al. 2022). These perceptual effects may vary by site, such as hands, arms, and thighs, and be shaped by both biological (Kern 2009) and experiential factors. A central question is whether these enhancements are shared across all DeafBlind individuals or are specific to those engaged in patterned exposure to a linguistic tactile system. Investigations into this question could provide deeper insight into how modality-specific experience (linguistic and otherwise) shapes language perception and processing.

From a grammatical perspective, the tactile modality may also give rise to novel constraints and possibilities. ASL exhibits flexible word order despite a canonical subject–verb–object base, with variation conditioned by verb morphology and information structure (e.g., Chen Pichler 2010, Cheng & Mayberry 2019, Sandler & Lillo-Martin 2006). Emmorey (2023) attributes some of this flexibility to the perceptual affordances in the visual modality. In protactile, managing tactile attention across multiple articulators (Edwards & Brentari 2020) may motivate distinct word orders and morphological strategies. We do not know yet if tactile interactions will favor foregrounding topical or spatial information earlier in the clause, capitalizing on the demonstratives described by Edwards & Brentari (2021), or create novel marking strategies for argument structure rooted in spatial or temporal sequencing. Understanding possibly newly emerging syntactic regularities in protactile will be crucial not only for understanding and documenting the language itself but also for expanding cross-modal typological theory.

Pragmatic and interactional norms represent another rich area for future work. Foundational aspects of conversation, such as backchanneling, signaling attention, taking and yielding turns, and the use of name signs, are being actively reconfigured within the tactile modality. As protactile continues to develop, future research could explore how such aspects are becoming grammatically encoded. Edwards (2022) points to the emergence of an intersubjective grammar in protactile that enables interlocutors to coordinate attention and reference without relying on vision or external guidance. This shift suggests that tactile interaction is not merely a pragmatic workaround but may involve the grammaticalization of engagement itself within the tactile modality. Future research might explore how these systems expand and shift over time, how they become routinized across contexts, and what new forms of interaction and alignment they afford. In doing so, we may better understand how grammars of engagement emerge in response to the tactile modality and its affordances and how they shape the evolving ecology of protactile conversation.

The emergence of protactile offers a rare opportunity to study how language develops under highly dispersed and socially complex conditions. Unlike many language communities, there is no central protactile-speaking population; DeafBlind people are often geographically isolated and must make intentional, sustained efforts to come together. Most DeafBlind children are born to sighted, hearing parents who are unfamiliar with any sign language (Gagne et al. 2023), let alone protactile, which complicates early exposure and transmission. Yet, despite these challenges, linguistic norms and practices are beginning to stabilize and spread. We are engaged in these intentional, sustained efforts to bring DeafBlind adults and DeafBlind children together, documenting the acquisition process. As more children enter protactile spaces, they play an important role in shaping the language, pushing it in new directions and contributing to its evolution. Understanding how these processes unfold in a new, tactile modality, without the infrastructure of a colocated community, will require longitudinal and ethnographically grounded research that captures both the social conditions and the linguistic innovations taking place.

Protactile also raises complex questions about the interface between language and technology. These questions differ substantially from those that involve spoken and signed languages. For example, because protactile is a tactile language coconstructed in physical space, it cannot be fully captured through conventional documentation methods such as writing or video (Edwards & Brentari 2020). This highlights the limitations of existing technologies and methodologies that fail to represent the tactile dimensions of the language. Furthermore, unlike spoken or signed languages, protactile cannot be transmitted remotely in a way that preserves its fundamentally tactile, coarticulated structure. Future research will need to take seriously the tactile, copresent nature of protactile by working within the physical conditions and community values that make the language possible.

Protactile offers linguists a compelling new frontier—one that pushes us to reconsider how language emerges and is sustained through tactile, copresent interaction. Its development highlights the grammaticalization of engagement through touch and raises important questions about documenting and transmitting language beyond visual or auditory means. Including the tactile modality in our theoretical lens not only deepens our understanding of protactile but also sharpens our broader theories of what language is and how it works.

## 5. ETHICS OF WORKING WITH THE PROTACTILE COMMUNITY

As we turn to the ethical considerations of working with the DeafBlind protactile community, it is important to first acknowledge our own positionality as researchers (Graham & Horejes 2017, Milner 2007). Positionality is not simply a checklist of demographic characteristics but rather a reflection of the relationships, experiences, and histories that shape how we engage with research (Boveda & Annamma 2023). By naming our positionality, we aim to make transparent the perspectives we bring to this work, recognizing that all research is shaped by the social and institutional positions of its authors. This practice not only clarifies our own subjectivity but also reinforces our accountability by acknowledging how our lived experiences have informed our research priorities and methodological choices.

Further, the questions protactile raises about the nature of language are inseparable from the social relationships and power dynamics involved in its study (Singleton et al. 2015). Because the language is still emerging within a small and often marginalized community, engaging with it responsibly requires more than curiosity—it demands reflexivity, accountability, and partnership (Graham & Horejes 2017, Singleton et al. 2014). We therefore begin this section by situating ourselves in relation to the work before outlining key ethical principles that we believe should guide research and collaboration with the DeafBlind protactile community.

### 5.1 Acknowledging Our Positionality

#### Deanna Gagne’s positionality (as of 2025):

I am a cisgender mixed-heritage female of Latine and Eastern European descent. I am hearing and sighted. I was born to deaf, signing parents and raised in a multigenerational home with extended family. The languages of my childhood household were ASL, English, and Spanish. My mother and grandparents immigrated to the United States from the Caribbean, and my father, a third-generation American, was born in the United States. My spouse is deaf, and I am a parent of three children: two hearing children (Codas, or Child of deaf adult) and one deaf child. All of my children are sighted. I currently work as an associate professor of linguistics at Gallaudet University. I have been working with the protactile community since 2020. While my life has been deeply shaped by my proximity to deaf and DeafBlind people, I acknowledge that this proximity does not automatically qualify me to research either ASL or protactile. I will never know what it is like to grow up or live as a deaf or DeafBlind person. I approach this work with humility, recognizing that my perspectives are shaped by my hearing and sighted privilege, my academic role, and my institutional affiliations. I aim to engage in reflexive, collaborative research that centers the lived experiences of those most impacted. These experiences guide how I understand and analyze the work, though I am always learning—from the communities I collaborate with and through my own ongoing reflection.

#### Hayley Broadway’s positionality (as of 2025):

I am a cisgender white DeafBlind female. My parents are hearing–sighted and learned how to communicate with me and my DeafBlind brother through Signed Exact English (SEE). I grew up attending a mainstream public school, until I decided I wanted to experience more as a Deaf person when I moved to a Deaf school in the later high school years. Around the same time,I learned of my Usher syndrome diagnosis. My language use evolved from English and SEE to ASL, then eventually some Tactile ASL later on. I finally experienced and embraced protactile when I was 29 years old, and it changed my life as a DeafBlind person and as an educator. I am a parent to two boys, one hearing–sighted Codba (Child of DeafBlind Adult) and one hearing–blind Codba. My spouse is also DeafBlind. Our household’s primary language is protactile, and my multilingual kids interact directly with each other through spoken English. I hold a bachelor’s degree in general and special education and a master’s in sign language education. While I consider myself a protactile expert, with years of experience in language acquisition education, protactile training education, and interpreting education, I strive to work closely with the protactile community to keep current on research, knowledge, and protactile language use.

### 5.2 Partnership Is Key

As interest in protactile language grows within the field of linguistics, it is essential to pause and reflect on the ethical responsibilities that come with working alongside the DeafBlind community. Protactile is not simply a novel modality or an underdocumented language deserving linguistic attention; it is a paradigmatic, epistemological shift grounded in tactile ways of being (Edwards 2024). Approaching research with the protactile community with the same frameworks used for visual or spoken languages risks replicating patterns of marginalization that have historically excluded DeafBlind individuals from leading the knowledge production about their own lives and languages.

To work ethically within this space, DeafBlind individuals—and in the case of protactile, those who are deeply immersed in the protactile community—must be positioned not merely as participants or consultants but also as coresearchers, cotheorists, and coleaders (Singleton et al. 2015). Sighted researchers, whether deaf or hearing, bring valuable skills and perspectives, but in this research, their role is to support, not to direct. This includes rethinking the traditional hierarchies of academic expertise; a PhD in linguistics does not outweigh the lived experience of a DeafBlind person with years of immersion in protactile language and its contributing movement. In all matters of analysis, interpretation, and methodological design, the intuitions of DeafBlind protactile users must guide the process.

This may appear to already align with broader movements of community-engaged or participatory research (Mikesell et al. 2013). However, protactile ethics requires more than consultation or inclusion. Ethical collaboration begins at the outset—during the formation of research questions, the design of the study, and the establishment of project goals. Plans made about a community, regardless of how well-intentioned, are inherently extractive (Kouritzin & Nakagawa 2018, Mervis 2024). Further, this work must be accountable to the protactile community—those who have undergone the deep linguistic and cultural shift that the protactile movement entails.

We recognize that not all DeafBlind individuals are embedded within the protactile movement and community. Some DeafBlind individuals navigate the world primarily through residual vision, spoken English, or tactile adaptations of sign language, to name a few approaches (see, e.g., van der Mark 2023). They may engage with protactile only when necessary or when the opportunity arises. They may clearly identify as DeafBlind and, as such, contribute meaningfully to many kinds of research and advocacy. It is important to understand the positionality and experience of all individuals who are contributing to research to effectively represent the intended linguistic and communicative contributions of the entire team and to avoid misrepresenting a particular experience and further marginalizing the community such work seeks to support.

The stakes here are not merely theoretical. As awareness of protactile grows, so does interest from researchers, educators, and practitioners across disciplines. This enthusiasm can be a powerful force for collaboration, but it also calls for care. Protactile is still an emerging community-rooted language. Because it is based on touch, which cannot be fully replicated remotely or virtually, it is simultaneously growing rapidly and slowly, spreading organically within DeafBlind networks while requiring sustained, in-person interaction.

For this reason, research and educational efforts must develop in tandem with the community and not outpace it. Because protactile is still taking shape, there is risk of unintentionally reproducing hierarchies where those with limited tactile experience are positioned as experts or gatekeepers. This is particularly true when research is conducted without meaningful, long-term relationships with protactile users. For linguists, this moment presents an opportunity to reflect on how the field approaches “discovery,” particularly when working with communities that have historically been marginalized or excluded from academic spaces (Terkourafi 2025). Rather than moving quickly to document or research, we can instead prioritize learning and building partnerships grounded in mutual respect and accountability. Developing this kind of trust also means taking a step back when asked. This may mean slowing down the pace of publication or relinquishing control over certain aspects of a project. It may also mean redirecting attention, such as giving authorship credit, to DeafBlind collaborators.

There is precedent for this kind of collaboration within the deaf community. The seminal work of William Stokoe is often cited as the beginning of modern ASL linguistics, but it could not have been accomplished without the contributions of his deaf collaborators, Dorothy Casterline and Carl Croneberg (Maher 1996). The future of protactile research will likewise depend on partnerships that center DeafBlind voices—not in symbolic terms but in the daily practices of coanalysis, cowriting, and coteaching.

Finally, ethical engagement also requires patience. The protactile community is not a static dataset waiting to be recorded. It is a living, growing network of people engaged in often difficult lived experiences. It is these very experiences that led to the protactile movement and language. Researchers must allow space for this growth to happen on its own terms. This includes recognizing that not every moment is a research opportunity and that, sometimes, the most ethical decision is to wait, to listen, or to step aside.

## 6. CONCLUSION

Protactile is not simply a new language; it expands our understanding of how language can emerge, be learned, and function in communities built around different sensory orientations. Developed through the everyday experiences and insights of DeafBlind individuals, protactile is a tactilefirst language that likely challenges conventional models of linguistic structure, interaction, and acquisition. As protactile vocabulary, phonology, and grammar continue to evolve, they offer rich insight into how human language can adapt and reinvent itself to align with its users.

At the same time, studying protactile requires new approaches to research and ethics. This work cannot follow the usual extractive models of academic inquiry. Because protactile is so closely tied to a way of being, the research process must center DeafBlind individuals—not only as participants but as coresearchers and cotheorists. Engaging with this process responsibly means building long-term relationships, practicing humility, and remaining grounded in the tactile, copresent realities that are the heart of the language and its community.

## Footnotes

1 Following the latest convention, protactile is written in lowercase except when it appears at the beginning of a sentence (for examples, see Edwards & Brentari 2021, Gagne et al. 2023).

2 In line with recent usage, we use the terms listeners and speakers to refer to language producers and receivers, regardless of modality; these terms denote production and reception, not specifically vocal or auditory processes. We use the term community of users to refer to the collective of listeners and speakers (for examples, see Edwards & Brentari 2020, 2021; Gagne et al. 2023).

3 DeafBlind refers to individuals with combined variable vision and hearing, encompassing a diverse community with a wide range of sensory experiences and communication preferences, including tactile, visual, and auditory modalities.

4 ASL does not have a standardized orthography. Consequently, sign language researchers typically use English glosses in all caps to represent the meanings of individual signs (Hochgesang 2022).

5 Video examples are available at https://osf.io/4wrgj/?view_only=f948f563ece245a988d5e842bc4b6cd2.
